# Seasonal variations in incidence and maternal–fetal outcomes of gestational diabetes

**DOI:** 10.1111/dme.14236

**Published:** 2020-02-05

**Authors:** C. L. Meek, B. Devoy, D. Simmons, C. J. Patient, A. R. Aiken, H. R. Murphy, C. E. Aiken

**Affiliations:** ^1^ Institute of Metabolic Science Addenbrooke's Hospital Cambridge UK; ^2^ Department of Clinical Biochemistry Cambridge University Hospitals Addenbrooke's Hospital Cambridge UK; ^3^ Wolfson Diabetes and Endocrinology Clinic Cambridge University Hospitals Addenbrooke's Hospital Cambridge UK; ^4^ Department of Chemistry Peterborough City Hospital Peterborough UK; ^5^ Department of Obstetrics and Gynaecology Rosie Hospital Cambridge University Hospitals Cambridge UK; ^6^ Norwich Medical School Bob Champion Research Building University of East Anglia Norwich UK; ^7^ Department of Women's Health King's College London London UK; ^8^ University Department of Obstetrics and Gynaecology University of Cambridge NIHR Cambridge Biomedical Research Centre Cambridge UK; ^9^ School of Medicine Western Sydney University Campbelltown NSW Australia; ^10^ LBJ School of Public Affairs University of Texas at Austin Austin TX USA

## Abstract

**Aims:**

To determine whether the neonatal and delivery outcomes of gestational diabetes vary seasonally in the context of a relatively cool temperate climate.

**Methods:**

A retrospect cohort of 23 735 women consecutively delivering singleton, live‐born term infants in a single tertiary obstetrics centre in the UK (2004–2008) was identified. A total of 985 (4.1%) met the diagnostic criteria for gestational diabetes. Additive dynamic regression models, adjusted for maternal age, BMI, parity and ethnicity, were used to compare gestational diabetes incidence and outcomes over annual cycles. Outcomes included: random plasma glucose at booking; gestational diabetes diagnosis; birth weight centile; and delivery mode.

**Results:**

The incidence of gestational diabetes varied by 30% from peak incidence (October births) to lowest incidence (March births; *P*=0.031). Ambient temperature at time of testing (28 weeks) was strongly positively associated with diagnosis (*P*<0.001). Significant seasonal variation was evident in birth weight in gestational diabetes‐affected pregnancies (average 54^th^ centile June to September; average 60^th^ centile December to March; *P*=0.027). Emergency Caesarean rates also showed significant seasonal variation of up to 50% (*P*=0.038), which was closely temporally correlated with increased birth weights.

**Conclusions:**

There is substantial seasonal variation in gestational diabetes incidence and maternal–fetal outcomes, even in a relatively cool temperate climate. The highest average birth weight and greatest risk of emergency Caesarean delivery occurs in women delivering during the spring months. Recognizing seasonal variation in neonatal and delivery outcomes provides new opportunity for individualizing approaches to managing gestational diabetes.

## What's new?

- Gestational diabetes (GDM) shows seasonal variation in hot climates, but there is no consensus on whether this impacts on neonatal or delivery outcomes.
- Birth weights and emergency Caesarean section rates vary seasonally in GDM‐affected pregnancies. The highest average birth weight and greatest risk of emergency Caesarean delivery occur when fewest births are complicated by GDM (March births).
- There are seasonal differences in GDM outcomes, and consideration should be given to the differing environmental, dietary and lifestyle factors faced by women with GDM throughout the year.

**What's new?**

- Gestational diabetes (GDM) shows seasonal variation in hot climates, but there is no consensus on whether this impacts on neonatal or delivery outcomes.
- Birth weights and emergency Caesarean section rates vary seasonally in GDM‐affected pregnancies. The highest average birth weight and greatest risk of emergency Caesarean delivery occur when fewest births are complicated by GDM (March births).
- There are seasonal differences in GDM outcomes, and consideration should be given to the differing environmental, dietary and lifestyle factors faced by women with GDM throughout the year.

## Introduction

Gestational diabetes (GDM) shows wide regional variations in prevalence (1–25%) in different settings around the world 1. The magnitude of this variation illustrates the importance of both genetic variation 2 and external environmental factors in the aetiology of GDM.

The likelihood of experiencing GDM depends on the individual's own underlying baseline glucose tolerance, which may be influenced by lifestyle 3 or genetic 4 factors, and the challenges posed by each pregnancy, for example, maternal adiposity, age and parity 5, 6. In addition to risk associated with individuals, however, there may also be factors in the general environment that influence the likelihood of GDM. Several recent reports suggest that GDM incidence varies with season in diverse settings including southern Europe 7, 8, 9, Brazil 10, Australia 11, 12, 13 and Canada 14, 15. These studies show that post‐load glucose values and incidence of GDM increase at higher ambient temperatures 7, 8, 10, 13, 14.

In the present study, we aimed to explore the association between time of year and GDM diagnosis in a cooler climate with less annual variation than many settings previously studied 7, 11, 13, 14. In Cambridge, UK, the average annual daily temperature is 11.2°C, with average monthly maximum temperatures between 7.3°C (January) and 22.8°C (July) 16. Previous work conducted in Brazil suggests that every additional degree Celsius increases the 2‐h glucose value obtained from an oral glucose tolerance test (OGTT) by an average of 0.07 mmol/l. However, a previous study in a cooler UK climate did not demonstrate any seasonal variation in diagnosis of GDM, albeit in a smaller cohort with a low baseline prevalence of GDM 17. If GDM incidence does vary seasonally in the relatively cool and invariant UK climate, then seasonal variation may be important across wider geographical areas than previously understood.

We hypothesized that not only the incidence, but also the neonatal and delivery outcomes of GDM could vary at different times of the year. This is important because seasonal variations in diagnosis rates may simply reflect differences in detection, whereas seasonal variations in outcomes would require serious consideration of modification of individualized treatment strategies at different times of year. Our objective was therefore to determine whether seasonal variation exists in the neonatal and delivery outcomes of GDM.

## Research design and methods

A cohort of 23 375 women who consecutively delivered singleton, live‐born infants at term (37–42 completed weeks of gestation), was identified over a 5‐year period (January 2004 to December 2008) in a single tertiary obstetrics centre in the UK. Women with pre‐existing diabetes were excluded from the analytical cohort. In cases where a women had more than one eligible birth at the centre during the study period, only the first was included.

All pregnant women were offered a random plasma glucose check at booking (usually performed at 11–16 weeks' gestation). In addition, women were screened for a second time at ~28 weeks with a 50‐g glucose challenge test; women with a glucose challenge test result >7.7 mmol/l were then referred for a 75‐g OGTT 18. Additional OGTTs were performed in later pregnancy on an *ad hoc* basis where clinically indicated.

The WHO 1999 criteria were used for GDM diagnosis until August 2007 (75‐g OGTT 0‐h ≥7.1 mmol/l, 2‐h ≥7.8 mmol/l) and the modified WHO 1999 criteria thereafter (75‐g OGTT 0‐h ≥6.1 mmol/l, 2‐h ≥7.8 mmol/l). Seasonal trends in pregnancy outcomes were not affected by the diagnostic criteria in use at the time, nor by the year of delivery within the 5‐year study period. Women with GDM were advised to follow a low glycaemic index diet and avoid excessive gestational weight gain. Women who had evidence of persistent hyperglycaemia were offered escalating treatment with insulin, metformin, or both, as per UK national guidelines 19.

For the OGTT, venous blood was collected using fluoride‐oxalate tubes and analysed using a hexokinase method (Dimension RXL MAX Clinical Chemistry System; Siemens Healthcare Diagnostics, Deerfield, IL, USA) in our accredited laboratory (Clinical Pathology Accreditation, UK).

Available maternal, neonatal and delivery characteristics included maternal age, maternal BMI (measured at first‐trimester booking), parity (collapsed into categories as 0, 1 and ≥2), and maternal ethnicity. Gestational age (measured by crown–rump length at first trimester ultrasonography) was recorded to the nearest week. Birth weight was measured to the nearest gram. Population‐specific birth weight centiles, adjusted for gestational age and fetal sex, were constructed for the study population. Mode of delivery was classified as spontaneous vaginal delivery, instrumental delivery, elective Caesarean section, or emergency Caesarean section.

The midwife assigned to the parturient recorded data regarding the pregnancy, delivery and neonate in an electronic maternity database as soon as possible after birth. This database is routinely maintained as part of hospital records and was not created specifically for study purposes. The database was regularly validated by a rolling programme of audits where the original case notes were checked against the information recorded in the database.

Weather data for the local area were recorded during the study period by the Cambridge Digital Technology Group weather station (located 3.3 miles from the hospital). These included temperature (degrees Celsius), dew point, humidity (%), atmospheric pressure (mBars), mean wind speed (knots), sunshine (h), rainfall (mm), and maximum wind speed (knots). All weather measurements were recorded at 30‐min intervals throughout the duration of the study (January 2004 to December 2008). Raw data were collapsed to average readings for each day of the year, which were then used as continuous numerical variables in logistic regression models in order to test whether adjusting for ambient conditions eliminated seasonal variation in GDM incidence, severity, or pregnancy outcomes.

We used logistic regression to model the factors influencing incidence and outcomes of GDM. We examined the risk of each outcome dependent on the day of the year (i.e. assigning all dates integers between 1 and 366) using generalized additive models in which all events were considered equivalent. Our models incorporated a non‐linear term for day of year on the risk of an adverse outcome, estimated using cubic splines. This model allowed us to avoid making any prior assumptions about the nature of the relationship between day of year and the risk of adverse outcomes. At the extremes of the annual cycle, values for consecutive dates lie within prediction intervals. Statistical significance of the non‐linear effect of day of year was assessed using a likelihood ratio test. Models were fitted for all pregnancies, and separate models were used for the subpopulation diagnosed with GDM. There was no independent effect of year of delivery on any of the models.

Findings were considered statistically significant at an α level of 0.05. Power calculations were performed by Monte Carlo simulation and demonstrated that the study had >90% power to detect 1% differences between groups at an α level of 0.05 for binary outcomes. All analyses were conducted using the R statistical software package version 3.5.1 20.

## Ethics

The study was approved as a service evaluation by the institution (‘The identification and management of gestational diabetes’; Project Record Number: 6232).

## Results

### Study population

Of the 23 735 pregnant women screened, 985 women (4.1%) were diagnosed with GDM. There was no significant variation in the total number of babies due or delivered throughout the year during the study period. Women with GDM were more likely to be older (*P*<0.001) and to have a higher BMI (*P*<0.001; Table 1) than euglycaemic women. Higher GDM risk was also associated with being of Asian (*P*<0.001) or black African (*P*=0.007) ethnicity. There was no significant effect of parity on GDM risk in unadjusted analysis. In adjusted analysis, increasing maternal age (*P*<0.001), maternal BMI at booking (*P*<0.001), lower parity (*P*<0.001), and being of Asian (*P*<0.001) or black African ethnicity (*P*=0.008) were all significantly associated with an increased risk of GDM (Table 2). In our cohort, 88% of GDM diagnoses were made at ~28 weeks. None of the demographic variables of the study population showed significant seasonal variation.

**Table 1:** Maternal and neonatal characteristics of population screened for gestational diabetes, by diagnosis

	All *N*=23 735	Normal glucose tolerance, *N*=22 641	Gestational diabetes, *N*=985	*P*
**Maternal characteristics**				
Maternal age, years	30.7 (±5.6)	30.61 (±5.7)	32.6 (±5.0)	<0.001
Parity, *n* (%)				
0	9123 (38.4)	8694 (39.2)	388 (39.4)	
1	9586 (40.4)	9168 (38.1)	399 (40.5)	
≥2	4979 (21.0)	4733 (22.5)	196 (19.9)	
Unknown	47 (0.2)	46 (0.2)	2 (0.2)	0.213
Ethnicity				
White European	21192 (89.3)	20280 (89.7)	815 (82.7)	
Asian	1249 (5.3)	1134 (5.0)	104 (10.6)	
Black	864 (3.6)	809 (3.6)	52 (5.3)	
Other	378 (1.6)	367 (1.6)	13 (1.3)	
Unknown	52 (0.2)	51 (0.1)	1 (0.1)	<0.001
Maternal BMI, *n* (%)				
<18.5 kg/m^2^	551 (2.3)	544 (2.4)	8 (0.8)	
18.5–24 kg/m^2^	11812 (49.8)	11423 (50.5)	396 (40.2)	
25–29 kg/m^2^	5068 (21.4)	4779 (21.1)	247 (25.1)	
30–34 kg/m^2^	1715 (7.2)	1573 (6.9)	110 (11.1)	
35–39 kg/m^2^	709 (2.9)	622 (2.7)	69 (7.0)	
≥40 kg/m^2^	342 (1.5)	288 (1.3)	38 (3.9)	
Unknown	3538 (14.9)	3412 (15.1)	117 (11.9)	<0.001
Random plasma glucose, mmol/l	5.8 (±1.4)	5.7 (±1.3)	7.8 (±1.9)	
OGTT, *n* (%)				
Yes	3603 (15.6)	2509 (11.1)	985 (100)	
No	20132 (84.8)	20132 (88.9)	0 (0)	
**Neonatal characteristics**				
Birth weight, g	3472 (±480.1)	3466 (±477)	3522 (±497)	<0.001
Gestation, weeks	39.6 (±1.2)	39.6 (±1.2)	39.1 (±1.3)	<0.001
Birth weight, median centile	50 (±28.6)	49 (±28.5)	57 (±29.0)	<0.001
Apgar score <7 at 5 min, *n* (%)				
No	23365 (98.5)	22289 (98.4)	968 (98.3)	
Yes	112 (0.5)	104 (0.5)	8 (0.8)	
Unknown	258 (1)	248 (1.1)	9 (0.9)	0.288
Admission to neonatal ICU at delivery, *n* (%)				
No	22956 (96.7)	21910 (96.75)	950 (96.4)	
Yes	772(3.3)	733 (3.2)	35 (3.6)	
Unknown	7 (0)	7 (0.1)	0 (0.0)	0.553
Mode of delivery, *n* (%)				
Spontaneous vaginal delivery	14550 (61.3)	14056 (62.2)	447 (45.4)	
Instrumental	3187 (13.4)	3038 (13.4)	139 (14.1)	
Emergency Caesarean section	3278 (13.8)	3030 (13.3)	202 (20.5)	
Elective Caesarean section	2680 (11.3)	2477 (11.1)	196 (19.9)	
Unknown	40 (0)	40 (0)	1 (0.1)	<0.001

ICU, intensive care unit; OGTT, oral glucose tolerance test.

Values are expressed as mean (± sd) unless otherwise indicated. *P* values are derived using Student's *t*‐test (unpaired, two‐tailed) for continuous numerical variables, and chi‐squared tests for discrete variables.

**Table 2:** Odds of gestational diabetes diagnosis by maternal characteristics in logistic regression analysis

Characteristic	Risk of diagnosis of GDM		
	Odds ratio	95% CI	*P*
Maternal age			
<25 years	0.33	0.25–0.43	<0.001
25–29 years	0.81	0.68–0.96	0.016
30–34 years	Reference		
35–39 years	1.35	1.15–1.58	<0.001
≥40 years	1.69	1.29–2.18	<0.001
Parity			
0	Reference		
1	0.90	0.76–0.95	<0.001
≥2	0.73	0.61–0.87	0.009
Ethnicity			
White European	Reference		
Asian	2.33	1.85–2.91	<0.001
Black	1.75	1.26–2.37	<0.001
Other	0.71	0.35–1.25	0.285
Maternal BMI			
<18.5 kg/m^2^	0.45	0.23–0.81	0.015
18.5–24 kg/m^2^	Reference		
25–29 kg/m^2^	1.47	1.26–1.70	<0.001
30–34 kg/m^2^	2.20	1.79–2.68	<0.001
35–39 kg/m^2^	3.61	2.78–4.65	<0.001
≥40 kg/m^2^	3.92	2.74–5.47	<0.001
Day of delivery (per week)	1.07	1.02–1.13	0.007
Average temperature at 28 weeks (per 5°C)	1.13	1.02–1.25	0.034
Average daily hours of sunshine at 28 weeks	0.99	0.96–1.02	0.440
Average 24‐h rainfall at 28 weeks	1.00	0.97–1.02	0.232
Average daily humidity at 28 weeks	1.01	0.99–1.03	0.451
Average daily maximum wind speed at 28 weeks	0.99	0.98–1.01	0.930
Average daily mean wind speed at 28 weeks	1.00	0.98–1.02	0.902
Average daily dew point at 28 weeks	1.00	0.98–1.02	0.424
Average daily atmospheric pressure at 28 weeks	0.99	0.98–1.01	0.961

GDM, gestational diabetes.

### Influence of weather measurements

After adjustment for maternal age, BMI, parity and ethnicity, there was no independent association with any weather measurement (including average daily hours of sunshine and 24‐h rainfall) other than average daily temperature with risk of GDM (Table 2). Higher temperatures on the day of screening (28 weeks) were significantly associated with the likelihood of undergoing a formal OGTT (odds ratio 1.21, CI 1.10–1.32 per additional 5°C; *P*<0.001), and with the likelihood of being diagnosed with GDM [odds ratio 1.13, CI 1.02–1.25 per additional 5°C; *P*<0.001 (Table 2)].

### Seasonal trends in gestational diabetes incidence

**Figure 1:**
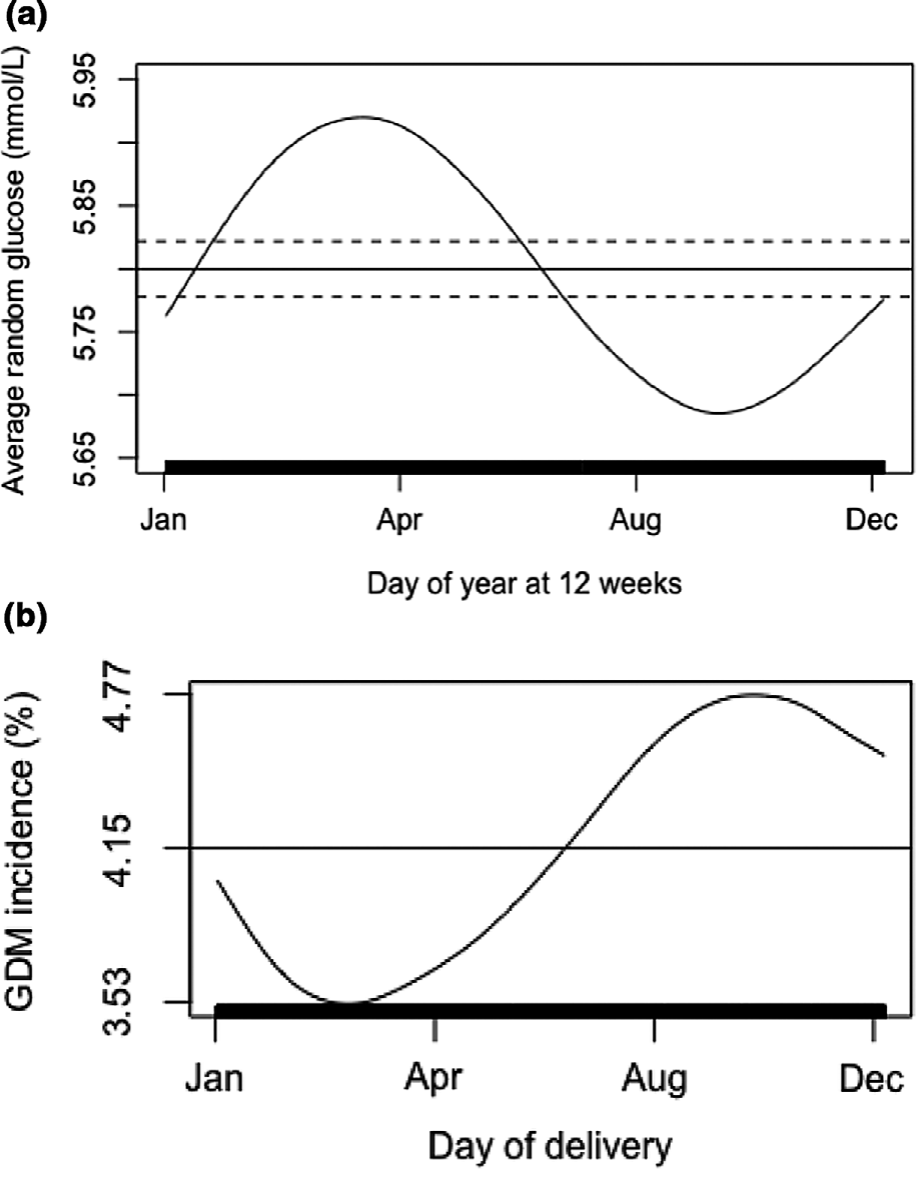
(a) Result of booking random plasma glucose dependent on day of screening (*P*<0.001). (b) Risk of diagnosis of gestational diabetes (GDM) dependent on day of delivery (*P*=0.031). *P* values refer to the significance of the non‐parametric trend in the relevant dependent variable across the annual cycle, derived from dynamic additive logistic regression models. *x*‐axis tick marks correspond in dates to 1 January (day 1), 30 April (day 120), 28 August (day 240) and 26 December (day 360), respectively. Vertical marks along the *x*‐axis represent individual observations. Horizontal line represents the mean risk level for the outcome; risks that are negative with respect to this line are therefore less likely than average, and those that are positive are more likely than average. Dashed lines represent the area within two standard errors of the mean for numeric variables only. Models are adjusted for maternal age, maternal BMI at booking, ethnicity, and parity.

Random plasma glucose levels at booking showed significant seasonal variation (*P*<0.001) (Fig. 1a). During spring (March–April) random plasma glucose concentrations were 15% above the population average and 15% below average in early autumn (August–September; Fig. 1a).

Using a non‐parametric model adjusted for maternal age, maternal BMI at booking, ethnicity and parity, there was significant (*P*=0.031) variation in the incidence of GDM over the year. Risk of GDM varied by 30% between the peak incidence (births during September/October) and lowest incidence (births during March; Fig. 1b). Maternal BMI at booking did not show any significant seasonal trend throughout the year.

### Obstetric and neonatal outcomes

Women with GDM had babies of higher average birth weight at slightly lower gestational ages compared to euglycaemic women [3601 g ± 524 g vs 3522 g ± 497 g; *P*<0.001 (Table 1)]. Delivery by Caesarean section, both elective (*P*<0.001) and emergency (*P*<0.001), was more likely in women with GDM (Table 1). There were no significant differences between mothers with GDM and euglycaemic women in the rates of low Apgar scores, or admission to the neonatal intensive care unit (Table 1).

**Figure 2:**
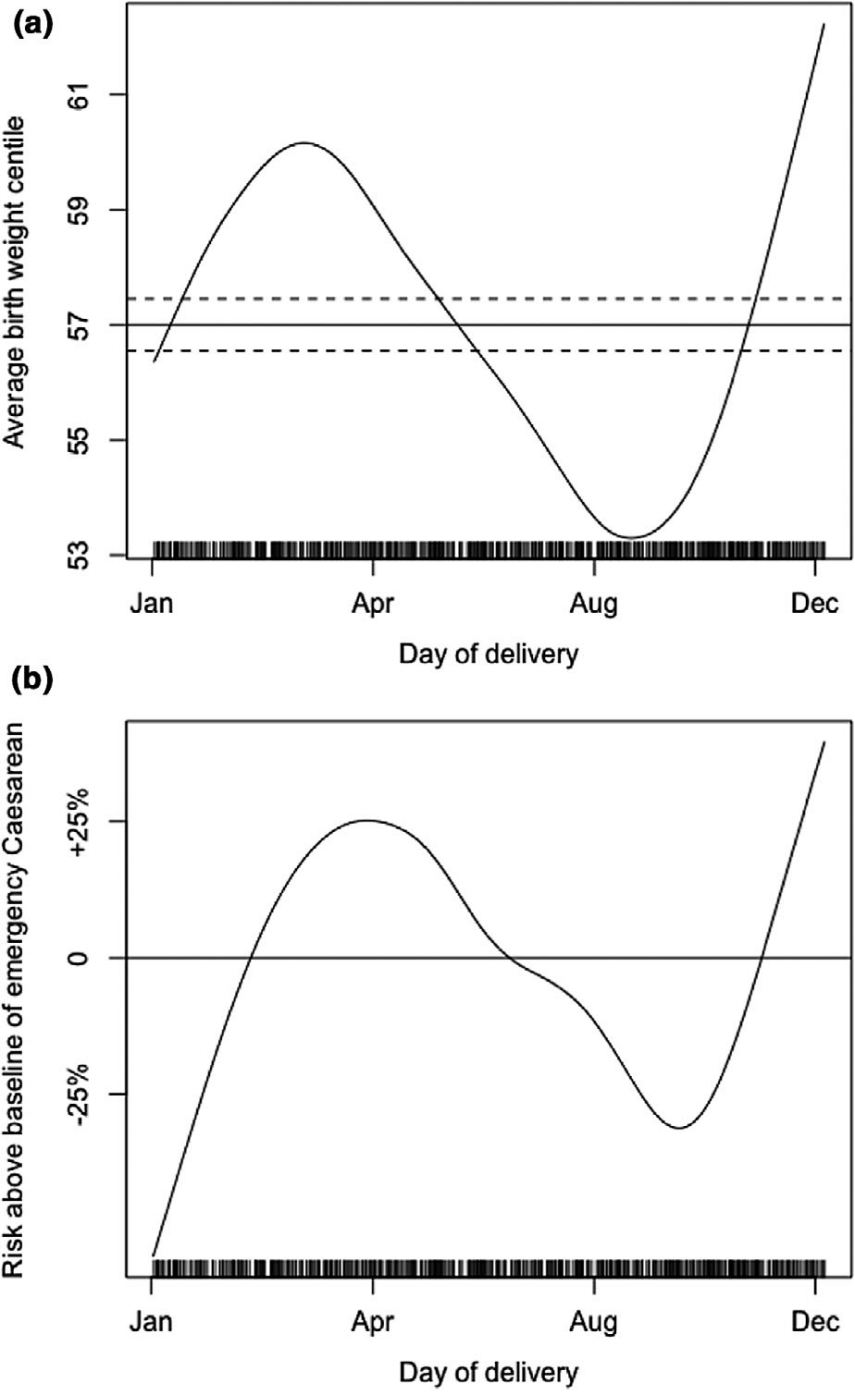
(a) Average birth weight centile dependent on day of delivery (*P*=0.027). (b) Likelihood of delivery by emergency Caesarean section dependent on day of delivery (*P*=0.038). *P* values refer to the significance of the non‐parametric trend in the relevant dependent variable across the annual cycle, derived from dynamic additive logistic regression models. *x*‐axis tick marks correspond in dates to 1 January (day 1), 30 April (day 120), 28 August (day 240) and 26 December (day 360), respectively. Vertical marks along the *x*‐axis represent individual observations. Horizontal line represents the mean risk level for the outcome; risks that are negative with respect to this line are therefore less likely than average, and those that are positive are more likely than average. Dashed lines represent the area within two standard errors of the mean for numeric variables only. Models are adjusted for maternal age, maternal BMI at booking, ethnicity and parity.

Babies born to mothers with GDM showed significant seasonal trends in birth weight centile (*P*=0.027) after adjustment for maternal BMI, ethnicity and parity (Fig. 2a). Babies born in summer to mothers with GDM were on average 54^th^ centile for gestational age, whereas those born to mothers with GDM in late December were on average 61^th^ centile. The percentage of babies born large for gestational age (>90^th^ centile) was 14% in August compared to 24% in December (overall *P* value for annual cycle = 0.047). In keeping with the seasonal variation in birth weight, there was also significant seasonal variation in the risk of delivery by emergency Caesarean section (*P*=0.038; Fig. 2b) in women with GDM. There was no seasonal variation in the risk of any other neonatal complications (unplanned admission to neonatal intensive care or Apgar score <7 at 5 min of life) born to mothers with GDM. There was no significant seasonal variation in any neonatal or delivery outcome in euglycaemic pregnancies.

## Discussion

In the present study, we observed marked variation in booking random plasma glucose levels and the incidence of GDM throughout the year in a large UK population. There was a close positive association between the ambient temperature at the time of screening and the likelihood of GDM. Furthermore, the risks of neonatal and delivery complications (high birth weight centile and emergency Caesarean delivery) varied significantly across the year in women with GDM. Strikingly, the risk of increased birth weight was highest at times of the year when incidence of GDM was lowest, and vice versa. Although GDM diagnosis is more likely when testing occurs at hotter times of year, adverse neonatal impacts are more likely when women experience GDM during colder times of year. This may be related to over‐diagnosis of GDM in hotter months, under‐diagnosis in cooler months, and/or to behavioural and lifestyle differences of pregnant women in colder months.

Our finding that glucose levels in pregnancy varied with ambient temperature is in keeping with a number of previous studies 7, 8, 10, 12, 14, 15 from various settings around the world. At hotter temperatures, the impact of a fixed glucose load may be greater due to reduced circulating plasma volume, leading to increased diagnoses of GDM. At increased ambient temperatures, physiological cooling mechanisms are activated that divert venous blood towards the skin, resulting in greater mixing of venous and capillary blood and altered concentrations of glucose 21. Our confirmation that significant seasonal variation occurs even in cooler ambient temperatures, such as those in the UK, has wider implications for developing testing methods that are more robust to seasonal variation.

There are numerous mechanisms that could potentially link the maternal–fetal outcomes of GDM to ambient temperatures. Opportunity for physical activity, which may be protective against developing GDM 22, is dependent on environment as well as socio‐economic opportunity. Particularly in environments where there are extremes of temperature, there may be reduced desire to exercise or fewer opportunities for physical activity during the hottest or coldest months 23. Diet composition and total calorie intake may also vary by season 24, 25. Social pressures to alter dietary patterns at different times of year may also contribute 24. In particular, the effect of holiday seasons, such as Christmas, on increased calorie consumption and weight gain are well documented 26. Despite previous work showing an association between maternal vitamin D status and risk of GDM 27, we found no independent association between daily hours of sunlight and GDM risk in our cohort.

We observed seasonal variation of up to 50% in the risk of delivery by emergency Caesarean section in mothers affected by GDM. Amongst populations of mothers with GDM, there is a known strong positive correlation between birth weight and risk of requiring emergency Caesarean section delivery 28, 29, but we are not aware of work that has examined this with regard to seasonal trends. This finding may have important implications for resource management within obstetric services.

The present study has several strengths, including its large well‐characterized dataset with detailed information on demographic variables and pregnancy outcomes, and the use of sophisticated statistical modelling. We used non‐parametric dynamic additive models as a powerful and flexible way to determine the risks of outcomes relative to baseline risk at any time point in the year while avoiding making any *a priori* assumptions about the risk/time relationship or introducing arbitrary time divisions within the annual cycle.

The study also has several limitations. Random plasma glucose testing was performed predominantly in community settings, where environments were not temperature‐controlled, samples were obtained at different times of day, and there were variable transportation times to the laboratory. Furthermore, multi‐step screening processes, which rely on measured glucose concentrations multiple times, may be more affected by variations in ambient temperatures compared to single‐step processes. This very large observational dataset also lacks detail on gestational weight gain in early pregnancy and family or previous obstetric history of diabetes, which may be important factors in determining GDM risk and outcome 30. Although our outcome models are adjusted for booking BMI values, it is plausible that weight gain during pregnancy may vary seasonally and this warrants further exploration. It is also possible that other factors, for example, maternal uptake of screening or maternal–fetal insulin production and sensitivity 15 may have seasonal trends which were not measured in this study.

New strategies for screening and diagnosis that are less affected by ambient conditions, in particular avoiding multi‐step processes, could potentially reduce spurious variation in GDM diagnosis rates. Pragmatic modifications to testing regimens should be considered to reduce excess seasonal variation in GDM diagnosis. It is an important clinical practice priority to ensure that conditions for GDM testing remain free of seasonal variation as far as possible. In particular, venesection should take place in a temperature‐controlled environment, at a defined time of day, and with minimal delay to sample processing.

Our results highlight that there may be an important unrecognized opportunity to improve neonatal and delivery outcomes in GDM by tailoring treatment strategies throughout the year. Investigating the possibility that neonatal outcomes could be improved with more intensive treatment of GDM during periods when the macrosomia risk is highest is an important research priority. Individualized treatment strategies should take account of the differing environmental, dietary and lifestyle factors faced by women with GDM at different times of year, for example, seasonally appropriate dietary modifications. The finding of seasonal trends in neonatal outcomes of GDM highlights the importance of considering women within their wider environmental context when planning an optimal treatment strategy with each individual.

## Funding sources

C.A. is supported by an Isaac Newton Trust/Wellcome Trust ISSF/ University of Cambridge Joint Research Grant. C.M. receives salary funding from the Diabetes UK Harry Keen Intermediate Clinical Fellowship (17/0005712). The funders had no role in study design, data collection, data analysis, manuscript preparation and/or publication decisions.

## Competing interests

C.M., B.D., D.S. and C.A. have no conflicts of interest to declare. H.R.M. has received honoraria as a member of the Medtronic European Advisory Board.
